# A multinational exploratory assessment of cancer knowledge among people with intellectual disabilities across European countries

**DOI:** 10.1186/s12939-026-02959-z

**Published:** 2026-07-29

**Authors:** Mohammed Elmadani, Simon Klara, Godfrey Mbaabu Limungi, Maha Besbes, Amer Mesmar, Osama Hamad, Lívia Tóth, Orsolya Máté

**Affiliations:** 1 Doctoral School of Health Sciences, Faculty of Health Sciences, University of Pécs, Vörösmarty Street 4, Pécs, H- 7622 Hungary; 2 Department of Epidemiology, Faculty of Public Health, University of El Imam El Mahdi, Kosti, Sudan; 3 Jamhuriya Research Centre, Jamhuriya University of Science and Technology, Mogadishu, Somalia; 4 Institute of Emergency Care, Pedagogy of Health and Nursing Sciences, Faculty of Health Sciences, University of Pécs, Pécs, Hungary

**Keywords:** Intellectual disabilities, Cancer knowledge, Health literacy, Cancer prevention, Health inequalities, Multinational study, Europe

## Abstract

**Background:**

People with intellectual disabilities (ID) experience significant health inequalities, including limited access to cancer-related information and poor cancer outcomes. Cancer knowledge is a key determinant of early detection and prevention; however, evidence from European countries remains limited. This study aimed to assess cancer knowledge among people with (ID) across European countries and to examine associated sociodemographic factors.

**Methods:**

A cross-sectional, multinational exploratory study was conducted between January 2025 and January 2026 across multiple European countries using a collaborative research network. A total of 896 adults with mild-to-moderate (ID) participated in this study. Cancer knowledge was assessed using an adapted, easy-to-read Cancer Awareness Measure covering three domains: warning signs (9 items), risk factors (11 items), and prevention (7 items). Data were analyzed using descriptive statistics, Spearman’s rank correlation, Mann–Whitney U test, Kruskal–Wallis test, and multiple linear regression.

**Results:**

All three knowledge domains demonstrated good internal consistency (Cronbach’s α = 0.802–0.929). Significant positive correlations were observed among knowledge of warning signs, risk factors, and prevention (ρ = 0.503–0.772, *p* < 0.001). Female participants had higher warning sign knowledge than male participants (*p* = 0.039). Participants living with their families had higher knowledge of warning signs and risk factors, whereas those in nursing homes showed higher prevention knowledge (*p* < 0.05). Significant differences were also observed across education, employment, and marital status (all *p* < 0.001), with higher education consistently associated with better knowledge.

**Conclusions:**

Cancer knowledge among people with (ID)varies across sociodemographic factors and is strongly interrelated across domains. The findings highlight the influence of structural and social determinants on health literacy in this population group. Targeted, accessible cancer education and inclusive health communication strategies are essential for reducing inequalities and improving cancer prevention across participating European settings.

**Supplementary Information:**

The online version contains supplementary material available at https://doi.org/10.1186/s12939-026-02959-z.

## Background

Cancer remains a leading cause of morbidity and mortality worldwide and represents a major public health challenge in Europe. According to the World Health Organization, cancer accounted for approximately 10 million deaths globally in 2020, and its burden continues to rise in both high- and middle-income countries [1]. In Europe, cancer is one of the leading causes of death, placing substantial pressure on healthcare systems and highlighting the importance of effective prevention and early detection strategies [2].

Early detection, timely help-seeking, and participation in preventive measures, such as screening, are strongly influenced by individuals’ knowledge and understanding of cancer. Awareness of symptoms, risk factors, and available health services is a key determinant of health-seeking behavior and plays a crucial role in facilitating early diagnosis and improving cancer outcomes [3, 4]. Limited cancer awareness is consistently associated with delayed presentation, lower screening uptake, and poorer prognosis [5].

People with intellectual disabilities (ID) experience persistent and well-documented health inequalities compared to the general population. Evidence indicates that people with ID are more likely to receive a cancer diagnosis at a later stage and experience poorer cancer-related outcomes, including higher mortality rates [6, 7]. These disparities are not solely explained by biological factors but are closely linked to social, structural, and systemic barriers within healthcare systems. Limited access to health information, communication difficulties, reliance on caregivers, and inadequate adjustments in healthcare settings contribute to delayed symptom recognition and reduced engagement with cancer prevention and screening services [8–11].

Cancer knowledge among people with ID is increasingly recognized as a critical yet under-researched area. Existing studies suggest that people with ID often have limited understanding of basic cancer concepts, such as the nature of cancer, common warning signs, and the purpose of screening [12, 13]. This knowledge gap may reduce their ability to recognize bodily changes, communicate their concerns, and participate meaningfully in health-related decision-making. While caregivers and healthcare professionals play an essential role in supporting health decisions, insufficient cancer knowledge among people with ID raises important ethical concerns regarding autonomy, informed consent, and equitable access to health information [14].

Despite the growing recognition of these challenges, most research on cancer knowledge among people with intellectual disabilities has been conducted within single countries or specific local contexts. Comparative multicountry evidence from Europe remains limited. This represents an important gap, given the diversity of healthcare systems, cancer prevention strategies, and disability support policies in European countries. Understanding cancer knowledge among people with ID across multiple settings can provide valuable insights into shared challenges and inform the development of inclusive and cross-national strategies for cancer prevention and health promotion.

Therefore, this study aimed to assess cancer knowledge among people with ID across 19 European countries using a multicountry cross-sectional design.

## Methods

### Study design and setting

This study employed a cross-sectional, multinational exploratory design to assess cancer knowledge among people with ID across European countries. Data collection was conducted between January 2025 and January 2026 through a collaborative network of academic institutions and international research partners. The study was conducted within a European research collaboration supported by the European Cooperation in Science and Technology (COST) framework. Invitations to participate were disseminated through COST-affiliated collaborators and partner institutions in multiple European countries. The participating institutions included, among others, Southeast Technological University, Radboud University Medical Center, University of York, Trinity College Dublin, University of Maribor, University of Novi Sad, and Yeditepe University. These institutions facilitated local coordination, recruitment, and the collection of data.

### Participants and sampling

This study targeted adults aged 18 years and older with mild-to-moderate ID. Participants were recruited from both institutional settings (nursing homes), and community-based settings, such as family homes, and supported living arrangements. Participants were classified as having mild-to-moderate intellectual disabilities based on records held by the participating institutions. Consistent with the DSM-5 framework, the severity of intellectual disability is determined primarily by adaptive functioning across conceptual, social, and practical domains rather than by IQ score alone, with mild and moderate levels typically corresponding to IQ ranges of approximately 50–70 and 35–50, respectively [15]. People with intellectual disabilities are a highly heterogeneous population, even within a single severity band. The AAIDD’s multidimensional model emphasizes that individuals with the same diagnostic classification can differ substantially in the type and intensity of support they require across communication, self-care, and community-participation domains [16]. This heterogeneity means that “mild-to-moderate ID” captures a wide range of communication abilities, independence, and dependency on caregivers or support staff. The present study did not collect data on ID subtype/etiology, standardized adaptive functioning scores, or level of support required, and severity classification was not independently verified across participating sites using a common diagnostic protocol. This is addressed further in the Strengths and Limitations section.

A non-probability convenience sampling approach was used in the participating countries. Recruitment was conducted through collaborating institutions, disability organizations, and professional networks associated with the COST partners. This approach was considered appropriate, given the absence of comprehensive sampling frames and the accessibility requirements of the target population.

Invitations were disseminated across a wide range of European countries in the study. However, participation depends on local engagement and recruitment feasibility. The final analytical sample consisted of 896 participants from multiple European countries. Country-level sample sizes varied, and not all the invited countries contributed data. Therefore, the study reflects an achieved multicountry sample rather than a predefined proportional allocation across the countries.

### Data collection procedure

**Fig. 1:**
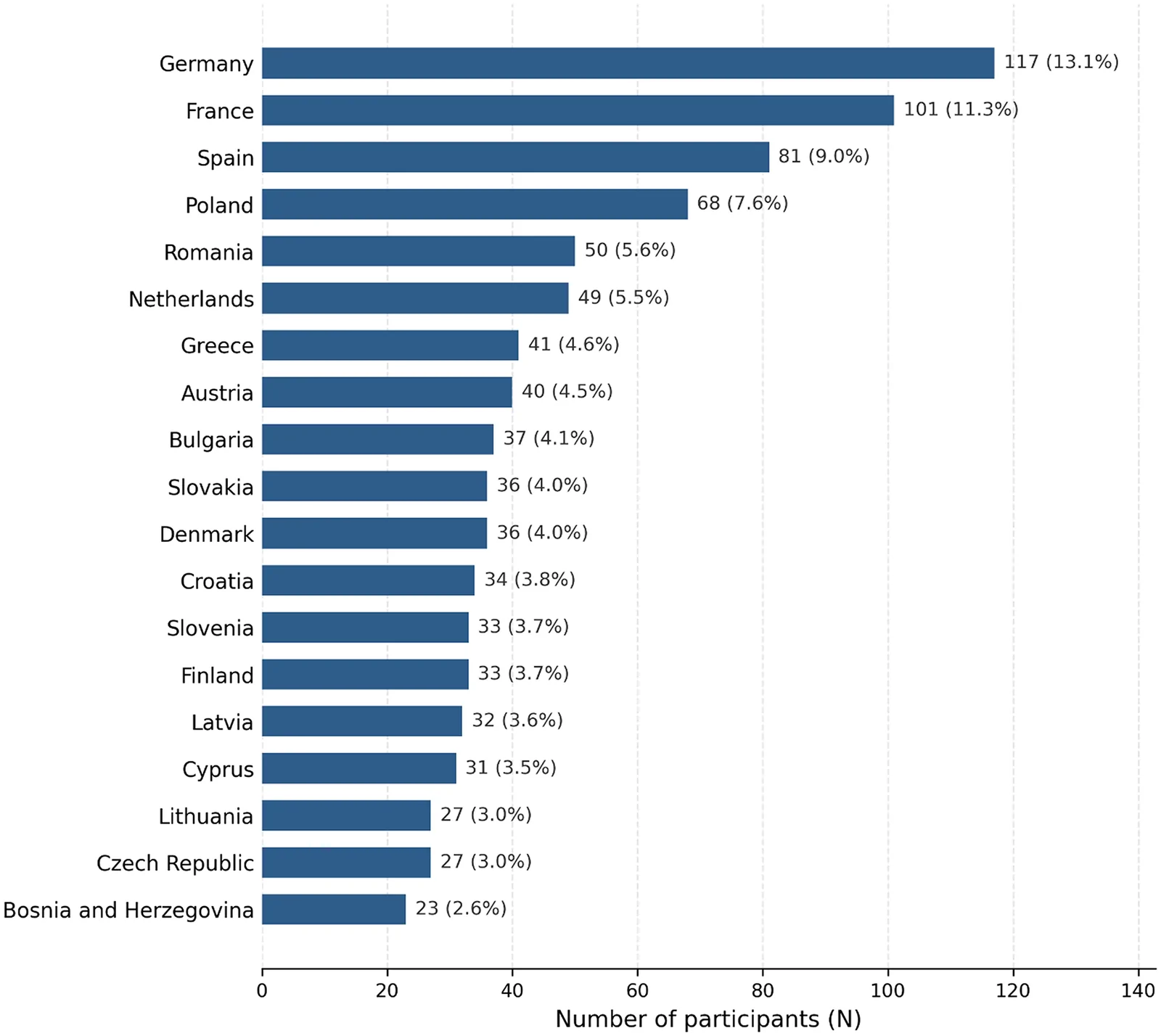
Number and percentage of participants by country in this multinational exploratory sample of people with intellectual disabilities (*N* = 896)

Data were collected using an accessible and easy-to-read questionnaire designed specifically for people with ID. The instrument was distributed through collaborating institutions and COST partners, whose members were already engaged in cancer prevention research among people with ID through the COST Action network. These local collaborators coordinated data collection in their respective countries via an online survey platform (Google Forms) and were provided with written guidance on the procedure, which was applied across 19 participating countries (Austria, Bosnia and Herzegovina, Bulgaria, Croatia, Cyprus, Czech Republic, Denmark, Finland, France, Germany, Greece, Latvia, Lithuania, Netherlands, Poland, Romania, Slovakia, Slovenia, and Spain). Recruitment occurred through nursing homes and family/community-based settings, with the number of participants per country ranging from 23 to 117 (Fig. 1).

To ensure accessibility and inclusivity, the questionnaire items were linguistically simplified, and the response options were standardized to be easy to understand. When participants required direct assistance to complete the questionnaire, it was most often provided by caregivers or family members rather than by research staff. These individual facilitators did not receive standardized training and were not issued specific instructions (e.g., to read items verbatim or avoid interpreting responses) to limit their influence on participants’ answers; no formal safeguards were implemented or documented to verify that this assistance did not affect responses. This distinction between the research-experienced local collaborators who coordinated the study and the caregivers/family members who directly assisted individual participants is an important nuance for interpreting the findings and is addressed further in the Strengths and Limitations section.

### Measures

#### Cancer knowledge assessment

Cancer knowledge was assessed using an adapted, easy-to-read version of the Cancer Awareness Measure (CAM) developed by Cancer Research UK [3]. The instrument was adapted to meet the communication and cognitive needs of individuals with intellectual disabilities through simplification of language, removal of complex or open-ended items, and the use of structured response formats. In the present study, cancer knowledge was evaluated across three domains.

The adapted CAM used in this study was originally developed and psychometrically validated in an independent study conducted with people with ID in Hungary [17]. This validation sample was separate from and not included in either the present multinational sample or the subsequent Hungarian cancer literacy study described below. Briefly, the original 47-item CAM [3] was adapted for cognitive accessibility through a multistage process involving item removal/simplification, expert panel review, cognitive interviews, and pilot testing [17].

The validated instrument was subsequently applied in a separate Hungarian study examining cancer literacy and help-seeking barriers among 350 people with ID [18]. The present study represents a distinct third dataset: a multinational sample of 896 participants recruited across 19 European countries, excluding Hungary, and is independent of both the original validation sample and the 350-participant Hungarian cancer literacy study. The same adapted CAM instrument (38 items across four subscales: Warning Signs, Risk Factors, Prevention, and Barriers to Seeking Help) was used across all three studies to ensure measurement consistency.

Hungary was excluded from the present multinational analysis and analyzed separately [18] because direct collaboration with the Hungarian Association for People with ID enabled substantially larger-scale data collection than was feasible through the standard COST Action network channels used across the other 19 countries. Including this disproportionately large, differently recruited sample within the multinational analysis would have skewed the country-level distribution and complicated cross-country comparability.

#### Knowledge of cancer warning signs

This domain included 9 items assessing the recognition of common cancer warning signs. Responses were coded as “Yes” = 1, and “No” = 0. A composite score was calculated by summing the item responses, yielding a total score ranging from 0 to 9, with higher scores indicating greater knowledge of cancer warning signs.

#### Knowledge of cancer risk factors

This domain consisted of 11 items that assessed awareness of cancer risk factors using a Likert-scale response format. Responses were coded as follows: strongly disagree = 0, disagree = 1, not sure = 2, agree = 3, and strongly agree = 4. The item scores were summed to produce a composite score ranging from 0 to 44, with higher scores indicating greater knowledge of cancer risk factors.

#### Knowledge of cancer prevention

This domain included seven items that assessed knowledge of preventive behaviors. Responses were coded as “Yes” = 1 and “No” = 0. A composite score was calculated by summing the item responses, yielding a total score ranging from 0 to 7, with higher scores reflecting better knowledge of cancer prevention.

#### Scoring approach

Higher scores in all domains indicated better cancer-related knowledge. Composite scores were computed by summing the item responses within each domain.

#### Reliability assessment

The internal consistency of each domain was assessed using Cronbach’s alpha.

### Statistical analysis

Descriptive statistics were used to summarize the participants’ characteristics and cancer knowledge scores. Continuous variables are presented as means and standard deviations (SD) or medians with interquartile ranges (IQR), as appropriate.

Of the initial pool of survey responses, 16 cases contained missing data on key study variables and were excluded prior to the analysis. The final analytic sample comprised 896 complete cases, and all analyses reported here used complete-case (listwise) analysis; no imputation was performed.

The internal consistency of the three knowledge domains was assessed using Cronbach’s alpha. The normality of the continuous variables was assessed using the Shapiro–Wilk test. The results indicated significant deviations from normality for all cancer knowledge domains (warning signs, risk factors, and prevention) (*p* < 0.001 for all). Therefore, non-parametric statistical tests were used in subsequent analyses. The Mann-Whitney U test was applied for comparisons between two groups, and the Kruskal-Wallis test was used for comparisons across more than two groups. The associations between the three knowledge domains were examined using Spearman’s rank correlation coefficient. All analyses were two-sided, and a p-value of less than 0.05 was considered statistically significant. Data were analyzed using the SPSS software (version 27).

### Methodological considerations

Because recruitment depended on the voluntary participation of collaborating countries and local feasibility, the final dataset represents an exploratory multicountry sample rather than a representative or population-proportional sample of European countries. Country-level differences in the sample size reflect variability in recruitment capacity and engagement. Therefore, the findings should be interpreted as exploratory and comparative rather than as nationally representative estimates.

## Results

### Participant characteristics

A total of 896 individuals with intellectual disabilities were included in the analysis (Table 1). The mean age of the participants was 38.69 years (SD = 16.06; range: 18–78 years). Most participants were female (60.0%, *n* = 538), while 40.0% (*n* = 358) were male. Regarding accommodation status, most lived with their families (76.9%, *n* = 689), whereas 23.1% (*n* = 207) resided in a nursing home.

The distribution of participants by country is presented in (Fig. 1). Sample sizes varied considerably across the 19 participating countries, ranging from 23 participants in Bosnia and Herzegovina to 117 in Germany. This reflects differences in the reach and capacity of collaborating institutions within the COST Action network rather than a proportionally stratified sampling design. Countries with larger, more established disability service networks and greater institutional engagement in the COST Action (e.g., Germany, France, Spain) contributed proportionally more participants, while countries with more limited network reach or later-joining partner institutions contributed smaller samples.

Formal statistical comparisons between countries were not performed for three reasons. First, several countries contributed relatively small samples (e.g., *n* = 23–30), which would provide limited statistical power for reliable between-country comparisons and would increase the risk of unstable or spurious estimates in any pairwise or multi-group analysis. Second, with 19 countries, a fully specified set of pairwise comparisons would incur a substantial multiple-comparisons burden, elevating the risk of Type I error unless corrected in ways that would further reduce power, given the smaller country-level samples. Third, and most fundamentally, because recruitment relied on convenience sampling through unevenly resourced institutional networks rather than nationally representative sampling frames, any observed between-country differences would likely reflect variations in recruitment mechanisms, participating institution types, and local service-system characteristics rather than genuine differences in cancer knowledge attributable to the national context. For these reasons, country-level statistical comparisons were considered beyond the scope of this exploratory study and are noted as a direction for future research using nationally representative or stratified sampling designs.

In terms of marital status, 37.2% (*n* = 333) were single/never married, 20.3% (*n* = 182) were partnered, 14.5% (*n* = 130) were divorced/widowed, and 28.0% (*n* = 251) preferred not to disclose their marital status. Educational attainment varied, with 44.0% (*n* = 394) reporting low education, 33.8% (*n* = 303) vocational education and training, and 22.2% (*n* = 199) higher education. Regarding employment status, 37.3% (*n* = 334) were not employed, 30.7% (*n* = 275) were employed, 12.6% (*n* = 113) were students, 2.3% (*n* = 21) were not in the workforce, and 17.1% (*n* = 153) preferred not to disclose their employment status.

**Table 1:** Sociodemographic characteristics of participants (*N* = 896)

Variable	Category	*n* (%)
Gender	Male	358 (40.0)
	Female	538 (60.0)
Age (years)	Mean ± SD	38.69 ± 16.06
	Range	18–78
Marital status	Single/never married	333 (37.2)
	Partnered	182 (20.3)
	Divorced/Widowed	130 (14.5)
	Prefer not to say	251 (28.0)
Education level	Low education	394 (44.0)
	Vocational education	303 (33.8)
	Higher education	199 (22.2)
Employment status	Not employed	334 (37.3)
	Employed	275 (30.7)
	Student	113 (12.6)
	Prefer not to say	153 (17.1)
	Not in workforce	21 (2.3)
Living arrangement	With family	689 (76.9)
	Nursing home	207 (23.1)

### Internal consistency of the cancer knowledge domains

The three cancer knowledge domains demonstrated good to excellent internal consistency. As shown in Table 2, Cronbach’s alpha was 0.917 for the warning signs domain, 0.929 for the risk factors domain, and 0.802 for the prevention domain, indicating that the adapted instrument had satisfactory reliability in this multinational sample.

**Table 2:** Internal consistency of cancer knowledge domains

Domain	No. of items	Cronbach’s α
Warning signs	9	0.917
Risk factors	11	0.929
Prevention	7	0.802

### Correlations between cancer knowledge domains

Spearman’s rank correlation analysis (Table 3) showed that all three cancer knowledge domains were positively and significantly correlated with one another. Knowledge of warning signs was strongly correlated with knowledge of risk factors (ρ = 0.772, *p* < 0.001) and moderately correlated with knowledge of prevention (ρ = 0.646, *p* < 0.001), respectively. Knowledge of risk factors was moderately correlated with knowledge of prevention (ρ = 0.503, *p* < 0.001).

**Table 3:** Spearman correlations between cancer knowledge domains

Variable	1	2	3
1. Warning signs	1	0.772***	0.646***
2. Risk factors	0.772***	1	0.503***
3. Prevention	0.646***	0.503***	1

### Differences in cancer knowledge by gender and accommodation status

Sex differences were observed only in the warning sign domain (Table 4). Female participants had significantly higher warning sign knowledge scores than male participants (U = 88,635.5, Z = -2.059, *p* = 0.039). No statistically significant sex differences were found in risk factor knowledge (*p* = 0.934) or prevention knowledge (*p* = 0.095).

Accommodation status was significantly associated with all three knowledge domains. Participants living with their families had significantly higher scores for warning signs (U = 54,889.0, Z = -5.126, *p* < 0.001) and risk factors (U = 57,860.5, Z = -4.156, *p* < 0.001) than those living in nursing homes. In contrast, participants living in nursing homes had significantly higher prevention knowledge scores (U = 63,473.5, Z = -2.454, *p* = 0.014).

**Table 4:** Differences in cancer knowledge by gender and living arrangement (Mann–Whitney U test)

Variable	Outcome	Median (IQR)	U	Z	*p*-value
**Gender**	**Warning signs**		88,635.50	-2.059	0.039
Male		3.0 (0.0–5.0)			
Female		3.0 (0.0–9.0)			
**Gender**	**Risk factors**		95,990.50	-0.083	0.934
Male		25.0 (22.0–28.0)			
Female		23.0 (22.0–33.0)			
**Gender**	**Prevention**		90,107.00	-1.669	0.095
Male		3.0 (1.0–4.0)			
Female		4.0 (1.0–7.0)			
**Living arrangement**	**Warning signs**		54,889.00	-5.126	< 0.001
Nursing home		0.0 (0.0–9.0)			
With family		3.0 (1.0–7.0)			
**Living arrangement**	**Risk factors**		57,860.50	-4.156	< 0.001
Nursing home		22.0 (22.0–28.0)			
With family		25.0 (22.0–30.0)			
**Living arrangement**	**Prevention**		63,473.50	-2.454	0.014
Nursing home		4.0 (2.0–6.0)			
With family		3.0 (1.0–6.0)			

Note. IQR = interquartile range (25th–75th percentile). *N* = 896 (male *n* = 358, female *n* = 538; nursing home *n* = 207, with family *n* = 689)

### Differences in cancer knowledge by marital status, education, and employment

Statistically significant differences in all three cancer knowledge domains were observed for marital status, education level, and employment status (Table 5). For marital status, significant differences were found in terms of warning signs (H = 199.596, df = 3, *p* < 0.001), risk factors (H = 110.703, df = 3, *p* < 0.001), and prevention (H = 171.137, df = 3, *p* < 0.001). Mean rank patterns suggested that participants who were divorced/widowed or partnered generally had higher knowledge scores than those who were single/never married, while those who selected prefer not to say tended to fall between these groups.

Education level was also significantly associated with the knowledge scores across all domains. Significant differences were identified in the knowledge of warning signs (H = 179.878, df = 2, *p* < 0.001), risk factors (H = 166.331, df = 2, *p* < 0.001), and prevention (H = 35.559, df = 2, *p* < 0.001). Participants with higher education consistently showed the highest mean ranks, followed by those with vocational education, whereas participants with low education had the lowest ranks in all domains.

Similarly, employment status showed significant differences in warning sign knowledge (H = 182.924, df = 4, *p* < 0.001), risk factor knowledge (H = 133.242, df = 4, *p* < 0.001), and prevention knowledge (H = 69.180, df = 4, *p* < 0.001). Participants who were employed and those classified as not in the workforce had the highest mean ranks across the domains, whereas those not employed generally had the lowest ranks. However, the very small size of the “not in workforce” group should be considered when interpreting these findings.

**Table 5:** Differences by marital status, education level, and employment status (Kruskal–Wallis test)

Variable	Outcome	*n*	Mean rank	H	df	*p*-value
**Marital status**	**Warning signs**			199.596	3	< 0.001
Divorced/Widowed		130	646.17			
Partnered		182	581.26			
Prefer not to say		251	390.38			
Single/never married		333	342.58			
**Marital status**	**Risk factors**			110.703	3	< 0.001
Divorced/Widowed		130	551.41			
Partnered		182	575.35			
Prefer not to say		251	426.05			
Single/never married		333	355.91			
**Marital status**	**Prevention**			171.137	3	< 0.001
Divorced/Widowed		130	534.99			
Partnered		182	625.08			
Prefer not to say		251	424.25			
Single/never married		333	336.50			
**Education**	**Warning signs**			179.878	2	< 0.001
Higher education		199	624.38			
Vocational education		303	481.22			
Low education		394	334.51			
**Education**	**Risk factors**			166.331	2	< 0.001
Higher education		199	568.05			
Vocational education		303	531.00			
Low education		394	324.67			
**Education**	**Prevention**			35.559	2	< 0.001
Higher education		199	509.87			
Vocational education		303	480.53			
Low education		394	392.87			
**Employment**	**Warning signs**			182.924	4	< 0.001
Not in workforce		21	766.07			
Employed		275	557.91			
Prefer not to say		153	501.67			
Student		113	445.85			
Not employed		334	314.99			
**Employment**	**Risk factors**			133.242	4	< 0.001
Not in workforce		21	842.71			
Employed		275	523.09			
Prefer not to say		153	511.16			
Student		113	385.79			
Not employed		334	354.81			
**Employment**	**Prevention**			69.180	4	< 0.001
Not in workforce		21	795.50			
Prefer not to say		153	510.01			
Employed		275	471.41			
Student		113	397.04			
Not employed		334	397.05			

### Item-level responses to cancer knowledge questions

Detailed item-level responses regarding cancer warning signs, risk factors, and preventive behaviors are presented in Supplementary Tables S1–S3.

### Warning signs

Overall, the recognition of cancer warning signs was moderate. The most frequently identified warning signs were unexplained bleeding (52.6%), persistent unexplained pain (49.0%), and unexplained lumps or swelling (47.1%). In contrast, lower levels of recognition were observed for difficulty in swallowing (31.3%) and unexplained weight loss (32.0%) (Supplementary Table S1).

### Risk factors

Awareness of cancer risk factors varied considerably across the items. The highest level of agreement was observed for smoking cigarettes, with 69.3% of participants agreeing or strongly agreeing that it increased cancer risk. Similarly, exposure to secondhand smoke and alcohol consumption were moderately recognized as risk factors. However, a substantial proportion of participants selected “not sure” for most items, particularly for HPV infection, low physical activity, and being overweight, indicating considerable uncertainty regarding these risk factors (Supplementary Table S2).

### Prevention behaviors

Knowledge of cancer prevention behaviors was relatively higher than that in other domains. Most participants correctly identified attending regular cancer screenings (66.6%) and moderating alcohol consumption (65.7%) as preventive behaviors. Similarly, engaging in regular physical activity (54.6%) and consuming fruits and vegetables (61.2%) were widely recognized by the participants. In contrast, lower awareness was observed regarding reducing animal fat intake (31.9%) and maintaining a healthy weight (37.2%) (Supplementary Table S3).

### Multiple linear regression results

Three multiple linear regression models were conducted (Supplementary Tables S4, S5, and S6) to examine the associations between sociodemographic characteristics (gender, accommodation type, marital status, education, and employment status) and knowledge of cancer in three domains: Warning Signs, Risk Factors, and Prevention. Single/never married, low education, not employed, male, and living with family were the reference categories.

All three models were statistically significant. The model predicting warning sign knowledge was significant, F (11, 884) = 49.55, *p* < 0.001, and explained approximately 38% of the variance (R² = 0.381, adjusted R² = 0.374). The model predicting risk factor knowledge was significant, F(11, 884) = 47.68, *p* < 0.001, R² = 0.372 (adjusted R² = 0.365). The model predicting prevention knowledge was significant, F (11, 884) = 37.62, *p* < 0.001, R² = 0.319 (adjusted R² = 0.310). Collinearity diagnostics indicated no concerns in any model (all VIF < 2.1, all tolerance > 0.49).

Across all three domains, partnered status (relative to single/never married), higher and vocational education (relative to low education), and most employment categories (relative to not employed) were associated with significantly higher knowledge scores (Tables S1–S3). Gender was not significantly associated with Warning Signs or Prevention knowledge but was significantly associated with Risk Factors knowledge, with women scoring lower than men (B = -1.16, 95% CI [-1.98, -0.34], *p* = 0.006).

Accommodation type showed a domain-specific pattern: nursing home residence was not significantly associated with Warning Signs (*p* = 0.316) or risk factor knowledge (*p* = 0.176), but was significantly and positively associated with prevention knowledge (B = 0.95, 95% CI [0.59, 1.31], *p* < 0.001), such that nursing home residents scored higher on prevention knowledge than those living with family, despite no corresponding advantage—and no disadvantage—in the other two domains.

## Discussion

This multinational exploratory study provides novel evidence on cancer knowledge among people with ID within this multicountry convenience sample. The findings indicate that the cancer knowledge domains of warning signs, risk factors, and prevention are strongly interrelated and vary significantly across sociodemographic characteristics, including gender, living arrangement, education, employment, and marital status. Overall, the results highlight persistent inequalities in cancer-related knowledge within this population and reinforce the need for targeted and accessible health education strategies.

The strong correlations observed between warning signs, risk factors, and prevention knowledge suggest that cancer knowledge in people with ID reflects a broader construct of health literacy, defined as the ability to access, understand, appraise, and apply health information in decision-making [19]. According to health literacy frameworks, individuals with higher health literacy levels are more likely to recognize symptoms, engage in preventive behaviors, and navigate healthcare systems effectively [20].

In this study, the interdependence of knowledge domains may indicate that people with ID rely on general, often fragmented health knowledge rather than structured or domain-specific understanding. This aligns with previous research showing that limited access to health information contributes to reduced health literacy in this population [21]. Therefore, the findings underscore the importance of developing interventions that go beyond information provision to support the comprehension, retention, and practical application of health knowledge.

The observed disparities in cancer knowledge across sociodemographic factors can be more effectively understood through the social model of disability, which conceptualizes disability as arising from societal and environmental barriers rather than individual impairments [22]. From this perspective, the lower levels of cancer knowledge observed among certain groups, such as individuals with lower education or those living in institutional settings, reflect systemic inequalities in access to information, communication and inclusive healthcare services.

Rather than attributing knowledge deficits solely to cognitive limitations, the findings highlight the role of informational exclusion, inaccessible communication, and inadequate and reasonable adjustments within healthcare systems [23–26]. These barriers limit opportunities for people with ID to engage with cancer-related information and participate in preventive health behaviors. This interpretation is consistent with evidence showing that healthcare systems often fail to provide appropriately adapted information for people with intellectual disabilities [8, 27, 28].

Gender differences were limited, with a significant difference observed only in warning sign knowledge, where female participants demonstrated slightly higher scores than male participants. This finding is consistent with studies in the general population, where women often exhibit greater awareness of cancer symptoms, possibly because of increased exposure to health campaigns and screening programs [29–31]. However, the absence of broader gender differences suggests that structural barriers affecting access to health information may operate similarly across genders in people with ID.

Living arrangements emerged as a key determinant of cancer knowledge, with participants living with their families demonstrating higher knowledge of warning signs and risk factors than those living in nursing homes [32, 33]. Within the social model of disability, this finding can be interpreted as reflecting differences in information environments [8, 34]. Family settings may provide more individualized communication and opportunities for informal learning, whereas institutional settings may limit access to tailored health education [12].

The finding that nursing home residents demonstrated higher prevention knowledge despite lower warning sign and risk factor knowledge is intriguing, and any explanation for this pattern must be regarded as speculative, since the present study did not directly assess the mechanisms underlying it. One possible, untested explanation is that nursing home residents may be exposed to structured routines or organized health-promotion activities that emphasize preventive behaviors specifically [35–37], while informal or incidental learning about warning signs and risk factors — which may depend more on family conversation, media exposure, or community-based information sources — could be comparatively limited in institutional settings. This interpretation is offered as a hypothesis for future research rather than as a confirmed explanation and should be tested directly, for example, through qualitative work exploring what forms of cancer-related information nursing home residents are actually exposed to. Regardless of the mechanism, the overall pattern suggests that institutional environments may not adequately support comprehensive health literacy development across all knowledge domains, highlighting the need for systematic integration of accessible health education within care settings. Education level was strongly associated with all three knowledge domains, with higher education linked to greater cancer awareness. This finding aligns with the established evidence that education is a key determinant of health literacy [38–41]. Individuals with higher educational attainment are better equipped to process complex health information and engage with health care systems.

Within the context of ID, educational inequalities may be compounded by limited access to inclusive and adapted educational resources. These findings reinforce the need for accessible health education interventions tailored to different cognitive abilities and learning needs.

Employment status was also significantly associated with cancer knowledge, suggesting that social participation plays an important role in shaping health and cancer literacy. Employed individuals may have greater exposure to social networks, information exchange, and structured environments that facilitate learning [42, 43]. Conversely, unemployed individuals may experience social isolation and reduced access to health information.

However, the highest scores observed among individuals classified as “not in the workforce” should be interpreted cautiously because of the small sample size. Overall, these findings highlight the importance of considering broader social determinants of health, including participation and inclusion, to understand health knowledge disparities.

Marital status was significantly associated with cancer knowledge, with partnered individuals generally demonstrating higher knowledge than single individuals. This may reflect the role of social support networks in facilitating access to health information and encouraging engagement with health care services [21, 44]. Partners and family members often act as intermediaries in communication with healthcare providers, particularly for people with ID. However, the high proportion of participants selecting “prefer not to say” limits the interpretability of these findings and highlights the need for caution when analyzing sensitive socio-demographic variables.

The regression results were broadly consistent with the bivariate patterns reported in the study: social connectedness (partnered status), formal education, and workforce engagement were the most consistent correlates of cancer knowledge across all three domains. This pattern aligns with the broader health literacy literature, in which social support and educational attainment have repeatedly emerged as key resources for health information access among people with ID [45–47].

The domain-specific effect of nursing home residence on prevention knowledge merits particular attention. One speculative explanation is that structured institutional settings may expose residents to more routine health promotion activities (e.g., scheduled health education sessions or awareness campaigns) that emphasize preventive behaviors specifically, without necessarily improving knowledge of warning signs or risk factors, which may depend more on informal information exposure through family or community networks. However, this interpretation is speculative and was not directly tested in the present study.

### Implications for policy and practice

The findings of this study have important implications for public health policies. From a health literacy perspective, there is a need to develop accessible, easy-to-read, and context-sensitive cancer education materials tailored to people with ID. From a social model perspective, interventions should address structural barriers, including inaccessible communication, lack of reasonable adjustments, and inequities in health care delivery.

Within the participating settings, these exploratory findings may inform ongoing efforts to promote inclusive healthcare, such as those pursued under the European Commission’s cancer strategies, while recognizing that confirmation in representative samples is required before broader conclusions can be drawn. Ensuring that cancer prevention programs are inclusive and accessible remains an important goal for reducing health inequities.

### Strengths and limitations

This study had several strengths, including its large sample size and multinational design, which provided a broad overview of cancer knowledge across diverse European contexts. The use of an adapted and accessible instrument further enhances the relevance of the findings.

However, this study has several limitations. This study relied on convenience sampling coordinated through COST-affiliated institutions, which limits generalizability; therefore, the findings should be interpreted as exploratory evidence from a multicountry convenience sample rather than as nationally or regionally representative estimates. While participants were characterized as having mild-to-moderate ID, further details on the type of ID, level of required support, and dependency on caregivers were not collected. As cancer knowledge may vary according to communication ability and degree of independence, this represents an important gap in sample characterization that future studies should address. Although the adapted, easy-to-read Cancer Awareness Measure demonstrated good internal consistency in the present sample, and construct/content validity evidence (including cognitive interviews and pilot testing with people with ID) was established during the instrument’s original adaptation and validation in an independent Hungarian sample, this validity evidence was not re-established specifically within the present multinational sample. Although the overall data collection procedure (online platform, written guidance to local collaborators) was applied consistently across participating countries, direct assistance during questionnaire completion was most often provided by caregivers or family members rather than trained research staff. Moreover, no standardized instructions or safeguards (e.g., verbatim item delivery, restrictions on interpretive input) were implemented or documented to minimize the influence of this assistance on participant responses. This may have introduced response variability that could not be assessed in the present analysis. Disability classification was based on the existing records of participating institutions rather than a standardized diagnostic verification procedure. Although multiple linear regression models were used to account for the interrelationships among sociodemographic predictors, residual confounding from unmeasured variables (e.g., communication ability, dependency needs, or country-level contextual factors not modelled here) cannot be ruled out. Finally, the cross-sectional design precludes causal inference, and the inclusion of some small or non-informative subgroups (e.g., participants who preferred not to disclose their marital or employment status) limits the interpretability of certain subgroup comparisons.

## Conclusion

This study demonstrates that cancer knowledge among individuals with intellectual disabilities is influenced by both individual and structural factors. Applying health literacy and social model perspectives highlights that knowledge gaps are not merely individual deficits but also reflect broader systemic inequalities. Addressing these disparities requires inclusive, accessible, and context-sensitive approaches to cancer education and healthcare delivery across participating European settings. 

## Supplementary Information

Below is the link to the electronic supplementary material.

Supplementary Material 1

## Data Availability

The datasets generated and/or analyzed in the current study are not publicly available owing to ethical and privacy considerations but can be made available from the corresponding author upon reasonable request.
