# Supplementary material for: A multinational exploratory assessment of cancer knowledge among people with intellectual disabilities across European countries
Source: Int J Equity Health. 2026 Jul 29;25:258. doi: 10.1186/s12939-026-02959-z (PMC13700228; doi:10.1186/s12939-026-02959-z)
Supplement: Supplementary file 1 [file 12939_2026_2959_MOESM1_ESM.docx]

**Supplementary Table S1. Recognition of cancer warning signs (n = 896)**

| **Warning sign item** | **Yes, n (%)** | **No, n (%)** |
| --- | --- | --- |
| Unexplained lump or swelling | 422 (47.1) | 474 (52.9) |
| Persistent unexplained pain | 439 (49.0) | 457 (51.0) |
| Unexplained bleeding | 471 (52.6) | 425 (47.4) |
| Persistent cough or hoarseness | 398 (44.4) | 496 (55.4) |
| Change in bowel or bladder habits | 369 (41.2) | 527 (58.8) |
| Difficulty swallowing | 280 (31.3) | 616 (68.8) |
| Change in a mole | 418 (46.7) | 475 (53.0) |
| Sore that does not heal | 397 (44.3) | 499 (55.7) |
| Unexplained weight loss | 287 (32.0) | 609 (68.0) |

*Note. The number and percentage of participants selecting each response option for each warning-sign item.*

**Supplementary Table S2. Awareness of cancer risk factors (n = 896)**

| **Risk factor item** | **Strongly disagree, n (%)** | **Disagree, n (%)** | **Not sure, n (%)** | **Agree, n (%)** | **Strongly agree, n (%)** |
| --- | --- | --- | --- | --- | --- |
| Smoking cigarettes | 33 (3.7) | 10 (1.1) | 232 (25.9) | 442 (49.3) | 179 (20.0) |
| Exposure to secondhand smoke | 33 (3.7) | 32 (3.6) | 432 (48.2) | 277 (30.9) | 122 (13.6) |
| Drinking >1 unit of alcohol daily | 23 (2.6) | 104 (11.6) | 413 (46.1) | 283 (31.6) | 70 (7.8) |
| Low fruit and vegetable intake | 48 (5.4) | 80 (8.9) | 415 (46.3) | 268 (29.9) | 85 (9.5) |
| Frequent red/processed meat consumption | 33 (3.7) | 104 (11.6) | 500 (55.8) | 214 (23.9) | 45 (5.0) |
| Being overweight (BMI >25) | 33 (3.7) | 65 (7.3) | 518 (57.8) | 212 (23.7) | 68 (7.6) |
| Sunburn during childhood | 21 (2.3) | 78 (8.7) | 467 (52.1) | 240 (26.8) | 88 (9.8) |
| Older age (>70 years) | 40 (4.5) | 75 (8.4) | 479 (53.5) | 230 (25.7) | 72 (8.0) |
| Family history of cancer | 29 (3.2) | 43 (4.8) | 480 (53.6) | 227 (25.3) | 117 (13.1) |
| HPV infection | 44 (4.9) | 57 (6.4) | 503 (56.1) | 201 (22.4) | 75 (8.4) |
| Low physical activity | 24 (2.7) | 69 (7.7) | 520 (58.0) | 200 (22.3) | 83 (9.3) |

*Note. The number and percentage of participants selecting each response option for each risk-factor item. BMI = body mass index; HPV = human papillomavirus.*

**Supplementary Table S3. Knowledge of cancer prevention behaviors (n = 896)**

| **Prevention behavior** | **Yes, n (%)** | **No, n (%)** |
| --- | --- | --- |
| Reduce animal fat intake | 286 (31.9) | 610 (68.1) |
| Maintain healthy weight | 333 (37.2) | 563 (62.8) |
| Eat ≥5 portions of fruit and vegetables daily | 548 (61.2) | 348 (38.8) |
| Limit sun exposure | 393 (43.9) | 503 (56.1) |
| Engage in regular physical activity | 489 (54.6) | 407 (45.4) |
| Moderate alcohol consumption | 589 (65.7) | 307 (34.3) |
| Attend regular cancer screening | 597 (66.6) | 299 (33.4) |

*Note. The number and percentage of participants selecting each response option for each prevention-behavior item.*

**Supplementary Table S4 Multiple Regression Predicting Warning Signs Knowledge**

| **Predictor** | **B** | **SE B** | **β** | **t** | **p** | **95% CI** | **VIF** |
| --- | --- | --- | --- | --- | --- | --- | --- |
| Female | -0.06 | 0.19 | -.01 | -0.33 | .744 | [-0.44, 0.32] | 1.09 |
| Nursing home | -0.26 | 0.26 | -.03 | -1.00 | .316 | [-0.77, 0.25] | 1.47 |
| Divorced/Widowed | 2.19 | 0.37 | .23 | 5.99 | <.001 | [1.47, 2.91] | 2.02 |
| Partnered | 2.56 | 0.30 | .30 | 8.70 | <.001 | [1.98, 3.14] | 1.72 |
| Prefer not to say (marital) | 0.22 | 0.28 | .03 | 0.78 | .439 | [-0.33, 0.77] | 1.95 |
| Higher education | 2.55 | 0.28 | .31 | 9.29 | <.001 | [2.01, 3.09] | 1.59 |
| Vocational education | 1.07 | 0.23 | .15 | 4.62 | <.001 | [0.61, 1.52] | 1.46 |
| Not in workforce | 4.48 | 0.68 | .20 | 6.62 | <.001 | [3.15, 5.81] | 1.28 |
| Prefer not to say (employment) | 2.25 | 0.30 | .25 | 7.51 | <.001 | [1.66, 2.84] | 1.55 |
| Student | 0.81 | 0.35 | .08 | 2.31 | .021 | [0.12, 1.50] | 1.65 |
| Employed | 1.09 | 0.27 | .15 | 3.99 | <.001 | [0.56, 1.63] | 1.95 |

*Note. N = 896. Reference categories: Male, With family, Single/never married, Low education, Not employed. F(11, 884) = 49.55, p < .001. R² = .381, adjusted R² = .374.*

**Supplementary Table S5 Multiple Regression Predicting Risk Factors Knowledge**

| **Predictor** | **B** | **SE B** | **β** | **t** | **p** | **95% CI** | **VIF** |
| --- | --- | --- | --- | --- | --- | --- | --- |
| Female | -1.16 | 0.42 | -.08 | -2.77 | .006 | [-1.98, -0.34] | 1.09 |
| Nursing home | -0.77 | 0.57 | -.04 | -1.36 | .176 | [-1.87, 0.34] | 1.47 |
| Divorced/Widowed | -1.88 | 0.79 | -.09 | -2.37 | .018 | [-3.44, -0.32] | 2.02 |
| Partnered | 4.62 | 0.64 | .25 | 7.23 | <.001 | [3.37, 5.88] | 1.72 |
| Prefer not to say (marital) | -1.86 | 0.61 | -.11 | -3.05 | .002 | [-3.05, -0.66] | 1.95 |
| Higher education | 5.49 | 0.60 | .31 | 9.21 | <.001 | [4.32, 6.66] | 1.59 |
| Vocational education | 5.34 | 0.50 | .34 | 10.67 | <.001 | [4.36, 6.33] | 1.46 |
| Not in workforce | 17.28 | 1.47 | .36 | 11.77 | <.001 | [14.40, 20.16] | 1.28 |
| Prefer not to say (employment) | 4.39 | 0.65 | .22 | 6.75 | <.001 | [3.12, 5.67] | 1.55 |
| Student | -0.15 | 0.76 | -.01 | -0.20 | .840 | [-1.65, 1.34] | 1.65 |
| Employed | 1.47 | 0.59 | .09 | 2.47 | .014 | [0.30, 2.63] | 1.95 |

*Note. N = 896. Reference categories: Male, with family, Single/never married, Low education, Not employed. F(11, 884) = 47.68, p < .001. R² = .372, adjusted R² = .365.*

**Supplementary Table S6 Multiple Regression Predicting Prevention Knowledge**

| **Predictor** | **B** | **SE B** | **β** | **t** | **p** | **95% CI** | **VIF** |
| --- | --- | --- | --- | --- | --- | --- | --- |
| Female | -0.15 | 0.14 | -.03 | -1.11 | .266 | [-0.42, 0.12] | 1.09 |
| Nursing home | 0.95 | 0.18 | .18 | 5.22 | <.001 | [0.59, 1.31] | 1.47 |
| Divorced/Widowed | 0.60 | 0.26 | .09 | 2.33 | .020 | [0.09, 1.10] | 2.02 |
| Partnered | 3.06 | 0.21 | .54 | 14.81 | <.001 | [2.65, 3.46] | 1.72 |
| Prefer not to say (marital) | 0.61 | 0.20 | .12 | 3.11 | .002 | [0.23, 1.00] | 1.95 |
| Higher education | 1.11 | 0.19 | .20 | 5.79 | <.001 | [0.74, 1.49] | 1.59 |
| Vocational education | 0.22 | 0.16 | .05 | 1.34 | .181 | [-0.10, 0.53] | 1.46 |
| Not in workforce | 3.61 | 0.47 | .24 | 7.61 | <.001 | [2.68, 4.54] | 1.28 |
| Prefer not to say (employment) | 1.05 | 0.21 | .17 | 4.99 | <.001 | [0.64, 1.46] | 1.55 |
| Student | 0.47 | 0.25 | .07 | 1.90 | .058 | [-0.02, 0.95] | 1.65 |
| Employed | -0.30 | 0.19 | -.06 | -1.59 | .113 | [-0.68, 0.07] | 1.95 |

*Note. N = 896. Reference categories: Male, With family, Single/never married, Low education, Not employed. F(11, 884) = 37.62, p < .001. R² = .319, adjusted R² = .310.*
